# Category learning in autistic individuals: A meta-analysis

**DOI:** 10.3758/s13423-023-02365-4

**Published:** 2023-09-06

**Authors:** Lena Wimmer, Tim M. Steininger, Annalena Schmid, Jörg Wittwer

**Affiliations:** 1 Department of Education, University of Freiburg, Rempartstr. 11, D-79098 Freiburg im Breisgau, Germany; 2 Faculty of Applied Psychology, SRH University Heidelberg, Heidelberg, Germany

**Keywords:** Autism, Category learning, Prototype formation, Meta-analysis

## Abstract

Learning new categories is a fundamental human skill. In the present article, we report the first comprehensive meta-analysis of category learning in autism. Including studies comparing groups of autistic and nonautistic individuals, we investigated whether autistic individuals differ in category learning from nonautistic individuals. In addition, we examined moderator variables accounting for variability between studies. A multilevel meta-analysis of *k* = 50 studies examining *n* = 1,220 autistic and *n* = 1,445 nonautistic individuals based on 112 effect sizes in terms of the standardized mean difference revealed lower-level category learning skills for autistic compared with nonautistic individuals, *g* = −0.55, 95% CI = [−0.73, −0.38], *p* < .0001. According to moderator analyses, the significant amount of heterogeneity, *Q*(111) = 617.88, *p* < .0001, was explained by only one of the moderator variables under investigation—namely, study language. For the remaining variables—namely, age, year of publication, risk of bias, type of control group, IQ of autistic group, percentage of male autistic participants, type of category, type of task, and type of dependent measure—there were no significant effects. Although hat values and Cook’s distance statistics confirmed the robustness of findings, results of Egger’s test and a funnel plot suggested the presence of publication bias reflecting an overrepresentation of disadvantageous findings for autistic groups. Objectives for future work include identifying additional moderator variables, examining downstream effects of suboptimal category learning skills, and developing interventions.

## Introduction

### The relevance of category learning for humankind

The ability to accurately classify persons, events, and objects into categories is central to adaptive human behavior (e.g., Ashby & Maddox, 2005). To illustrate, categorizing a wild plant as edible typically leads to behavior different from that following upon categorization as poisonous, with possibly devastating consequences of incorrect classification. Categories also support pivotal processes including comprehension, learning, inference generation, explanations as well as language and communication (for an overview, see Medin & Rips, 2005). Hence, learning new categories can be considered a fundamental skill (Kruschke, 2005). Given the importance of categories for everyday behavior, it is vital to identify groups of people for whom category acquisition poses challenges in order that adequate learning support can be provided. Autistic individuals may be such a group as suggested by theoretical accounts of autism (Mottron et al., 2006; O’Riordan & Plaisted, 2001; Pellicano & Burr, 2012; van Boxtel & Lu, 2013; Van de Cruys et al., 2014). According to ICD-11 criteria (World Health Organization, 2019), autism spectrum disorder is a neurodevelopmental disorder defined by difficulties in social interaction and communication as well as restrictive, repetitive, and inflexible behaviors and interests (for other conceptualizations of autism, see Milton, 2019). In the present article, we report the first comprehensive meta-analysis of category learning in autistic individuals.

### Theoretical models of autism and category learning

Several theoretical models predict that autistic individuals differ in category learning from nonautistic individuals: The *enhanced discrimination hypothesis* (O’Riordan & Plaisted, 2001) postulates that autistic individuals compared with nonautistic individuals have an advanced ability to discriminate between perceptual stimuli. Hence, when learning new categories, autistic individuals can be expected to build categories with more narrow bounds and less variable category members than nonautistic individuals. This could be an advantage when homogeneous categories (e.g., the category of song birds) are to be learned, but problematic when the category of interest is diverse (e.g., the category of vertebrates).

The *enhanced perceptual functioning model* (Mottron et al., 2006) assumes that autistic persons differ from nonautistic persons through more fine-grained discrimination in low-level perception, superior pattern recognition in medium-level perception, and greater independence of lower-level perception from processes of top-down control. Similar to the enhanced discrimination hypothesis (O’Riordan & Plaisted, 2001), a possible prediction of this model anticipates disadvantages for learning diverse categories in addition to categories whose acquisition requires top-down control. The latter may be exemplified by categories for which perceptual resemblance to in fact conceptually distinct categories is a misleading cue for category membership. For example, whales are perceptually similar to fish, yet belong to the category of mammals.

Furthermore, the *HIPPEA* (*high, inflexible precision of prediction errors* *in autism*; Van de Cruys et al., 2014; see also the *predictive coding* theory of autism: Pellicano & Burr, 2012; van Boxtel & Lu, 2013) *model* conceptualizes autism as a condition of exceptional information processing. It is argued that humans navigate the world based on predictions. If prediction errors occur, it is essential to recognize which errors are serious and need to be learned from and which errors are not essential and can or even need to be ignored to facilitate abstract inferences. The HIPPEA model posits that autistic individuals give inflexibly high weight to prediction errors without sufficient distinction between critical and negligible mistakes. Hence, when learning new categories, this oversensitivity to prediction errors might make it difficult to identify stimulus features or relations between features that specify category membership. This should result in general disadvantages in category acquisition for autistic compared with nonautistic individuals. Overall, difficulties in acquiring at least some types of categories are proposed by the above-mentioned models of autism (for an overview of further theoretical approaches, see Mercado et al., 2020). To decide whether this assumption is warranted, a thorough review of the empirical evidence would be desirable.

### Previous reviews of category learning in autistic individuals and open questions

Three review articles relevant to the category learning skills of autistic individuals have been published in recent years: First, Patry and Horn (2019) summarized the evidence regarding prototype formation, categorization, and schema development in autistic individuals based on a systematic review of 23 studies published between 1980 and 2018. Second, Mercado et al. (2020) focused their narrative review on autism-related acquisition of perceptual categories and how it affects cognitive development and social symptoms of autism. Third, Vanpaemel and Bayer (2021) suggested in a narrative review of prototype-based category learning in autistic individuals that the heterogeneity in findings could be related to differences in research methodology.

Even though all of these narrative reviews (Mercado et al., 2020; Patry & Horn, 2019; Vanpaemel & Bayer, 2021) make an important contribution to research on category learning in autistic individuals, so far, a comprehensive and quantitative overview of this topic is still lacking. This is because the reviews either focused on how one type of *mental representation* is built up, in particular by looking at prototype formation (see Patry & Horn, 2019; Vanpaemel & Bayer, 2021), or because a single *type of categories*, namely perceptual categories, was taken into account (see Mercado et al., 2020). A thorough synthesis of the literature should ideally cover all kinds of mental representations, types of categories, and category learning tasks, as well as address heterogeneity between studies.

A great deal of research into categorization has focused on the question how categories are stored within the human mind (Medin & Rips, 2005). In short, the current evidence base does not suggest that humans rely on a single, but instead on several types of mental representation, including a mental representation in terms of rules that specify necessary and sufficient conditions for all category members (e.g., Bruner et al., 1956), as bunches of characteristic features whose average constitutes the prototype (e.g., Rosch & Mervis, 1975), or as individual instances, or exemplars, each attached with a category label (e.g., Brooks, 1978). Thus, a comprehensive account of category learning should incorporate more than one of type of mental representation, that is, go beyond prototype formation—the focus of Patry and Horn’s (2019) and Vanpaemel and Bayer’s (2021) syntheses.

Another line of research addresses the question whether there are different types of categories. A classification of categories widely acknowledged in categorization research is the distinction between isolated (e.g., Bott et al., 2006) and interrelated (e.g., Hetzroni & Shalahevich, 2018) concepts (Medin et al., 2000; a concept can be understood as the mental representation of a category; Markman & Ross, 2003). According to Goldstone (1996), along the isolated-interrelated continuum a category can be said to be the more interrelated the more it is affected by other categories. For instance, color is an example of an isolated category since it can be considered independently from other categories (see Shu et al., 2001). In contrast, the function of an object provides an example of interrelated categories since it is defined by the use of an object for a specific purpose and so cannot be regarded in isolation (see Field et al., 2016). In sum, a comprehensive look at category learning may benefit from addressing more than one type of category, for instance, by covering both isolated and interrelated categories. As opposed to this, the review by Mercado et al. (2020) is confined to perceptual categories. Our literature search suggests that so far no study has directly contrasted the processing of isolated versus interrelated categories in autistic persons. However, individual studies within autism research provide information as to whether isolated or interrelated categories were investigated. Hence, in the present meta-analysis we included type of category (isolated vs. interrelated) as moderator variable varying between studies.

Moderator analyses as the one implemented for type of category present a specific advantage of meta-analyses since they afford the possibility to not only quantitatively synthesize findings but to also account for heterogeneity between studies, a known characteristic of empirical research on category learning in autistic individuals (e.g., Dovgopoly & Mercado, 2013). Based on empirical findings, several further variables suggest themselves as potential moderators: An important determinant of category learning is the type of task used within research. Ashby and Maddox (2005) differentiate between rule-based, information-integration, and prototype distortion tasks (they also mention the weather prediction task; however, as our literature search suggests that this task type has not been used in autism research, we will not elaborate on it). In *rule-based* tasks, categories can be acquired through deliberate reasoning. Typically, the ideal classification or learning strategy can easily be verbalized (Ashby et al., 1998). In the Wisconsin Card Sorting Test (WCST; used by, e.g., Park et al., 2014, see also Table 2), for instance, four pictures resembling playing cards are presented. The cards depict geometric shapes varying in color, shape, and number. Trial by trial, participants need to assign an additional card to only one of the four stimulus cards, although frequently more than one match, each on a different dimension, would be possible (e.g., the sorting card matches one of the stimulus cards in shape color, and another stimulus card in shape number). Feedback informs about the accuracy of each sort. This supports a rule-based approach in which participants systematically try out the different sorting dimensions until they can establish a link between sorting dimension and accuracy. This strategy is typically easy to articulate verbally. In contrast, optimal performance in *information-integration* category learning tasks is achieved when information from at least two stimulus aspects or dimensions is integrated before a task-relevant decision is made (Ashby & Gott, 1988). Usually, the strategy leading to ideal performance is difficult to describe verbally or cannot be verbalized at all (Ashby et al., 1998). In the study by Plaisted et al. (1998), for example, participants had to learn how to accurately assign eight circle configurations to one of two categories. Each of these categories was defined by two rules, one of which stated that the position of certain circles was fixed, whereas the other rule indicated that the position of the remaining circles was irrelevant for category membership. Optimal performance in this case benefits from predecisional information integration and may hardly be verbalizable (there is also evidence to suggest that articulability supports category acquisition; see Zettersten & Lupyan, 2020). Finally, the categories to be learned within *prototype distortion* tasks are devised by first producing a stimulus that serves as the prototype (Posner & Keele, 1968, 1970). Subsequently, the remaining category members are constructed by randomly distorting the prototype. An example of this task type was used by Gastgeb et al. (2009): Participants were presented with line-drawn faces that differed from a prototypical face by variations in face length, nose length, nose width, and interocular distance. The relation between type of category and type of category learning task has not received a great deal of attention within research. Hence, looking at type of category and sort of category learning task as separate factors seems justifiable.

Furthermore, two different types of dependent measures are commonly used in research on category learning, namely accuracy (e.g., Potrzeba et al., 2015) and response time (e.g., Rumsey, 1985). It seems therefore worth exploring whether the difference between autistic and nonautistic individuals varies depending on these types of dependent measure.

A set of further potential moderators is related to participant characteristics: language of participants in view of well-known relationships between category formation, concepts, and language (Bowerman & Levinson 2001; Hahn & Cantrell, 2012; Sloutsky & Deng, 2019), age of autistic individuals, since category learning is likely to develop over many years (Patry & Horn, 2019), general cognitive ability, which has been proposed as a factor underlying interindividual differences in category learning, also in autistic individuals (see Dovgopoly & Mercado, 2013), and percentage of male research participants given that autism is more frequent in males than females (Zeidan et al., 2022) so that an unrepresentative distribution of this variable could obliterate the specific characteristics of category learning in autistic individuals. Furthermore, though not a participant characteristic, the year in which studies are published could act as a moderator variable in light of an increase in measured prevalence of autism over the last decades (Zeidan et al., 2022).

Finally, heterogeneity between studies may go back to differences in research methodology. This could be picked up by study quality, for example, by way of validating the ASD diagnosis (Desaunay et al., 2020).

### The present meta-analysis

We carried out a meta-analysis on category learning in autistic individuals across all kinds of mental representations and all types of categories to give a thorough overview and to explain the earlier observed between-study heterogeneity.^1^ Our primary research question concerned whether and to what extent autistic individuals differ in category learning from nonautistic individuals. Based on earlier reviews (Mercado et al., 2020; Patry & Horn, 2019; Vanpaemel & Bayer, 2021), we predicted that autistic persons would show lower performance levels than nonautistic persons. In addition, we aimed to identify moderator variables accounting for variability between studies. To this end, the following variables were considered: type of category (isolated vs. interrelated), type of category learning task (information-integration vs. prototype distortion vs. rule-based), type of dependent measure (accuracy vs. response time), study language, age of autistic individuals, type of control group (matched vs. not matched on IQ), IQ of autistic individuals, percentage of male research participants, year of publication, and risk of bias (validation via Autism Diagnostic Interview - Revised (ADI-R) and Autism Diagnostic Observation Schedule (ADOS) vs. validation via ADI-R or ADOS vs. other).

Regarding study design, we did not include investigations using a single-subject design due to associated limitations in quantitative data analysis that can impede meta-analytical synthesis (Sandbank et al., 2020) and studies lacking a comparison group of nonautistic individuals since these are restricted with regards to autism-specific assertions. Studies were integrated based on effect sizes in terms of the standardized mean difference, as Hedges’ *g*. Heterogeneity was determined by means of χ^2^ (*Q*) and Higgins *I*^*2*^ tests. There was no protocol for this synthesis.

## Materials and method

### Transparency and openness

We adhered to the MARS guidelines for meta-analytic reporting (Appelbaum et al., 2018). All meta-analytic data, analysis code, and research materials (including our coding scheme) are available online (https://osf.io/gtj2p/). This review project was not preregistered.

### Selection criteria

We selected studies with the following inclusion criteria:

1. The study was published in 1970 or later.
2. The full text is written in English.
3. The study compared at least one group of autistic individuals with at least one group of nonautistic individuals.
4. The study investigated category learning.

The only exclusion criterion we applied was:

1. The study implemented a single-subject design.

Reports that did not contain sufficient information to judge eligibility were excluded from analysis. If reports did not include sufficient information for analysis, authors with findable current contact details were contacted. In case the necessary details could still not be obtained, reports were excluded.

### Search strategy

In order to detect all studies of interest, we performed a literature search using ERIC (via Institute of Education Sciences), PsycInfo (via EBSCOhost), MEDLINE (via PubMed), and Web of Science (via Clarivate). The following expression of search terms was used for each database, with no specific search fields selected: (autis* OR asd OR pdd OR asperg*) AND (categ* OR concept* OR prototype OR schema OR script). This database search was carried out on January 5, 2021, and updated on January 13, 2022. In addition, we conducted a manual search and inspected the reference lists of the reviews by Patry and Horn (2019), Mercado et al. (2020), and Vanpaemel and Bayer (2021) as well as the reference lists of the 10 most recent included articles retrieved from the database search. For five reports where full texts were not accessible, authors were emailed. Two of them responded and supplied their full text. In addition, 14 authors were contacted since full text reports did not state all statistics needed for effect size calculation. Of these, three researchers replied, with one providing the requested details.

### Data extraction

A group of five researchers (three of the authors plus two research assistants) screened titles and abstracts. Each of these researchers initially screened a distinct subset of the database hits. To further assess eligibility of the entries remaining after screening, each full text was read by at least one researcher. Ambiguous cases that could not be excluded with certainty were reassessed by at least one other researcher, and conflicting assessments were resolved through mutual discussion. The final set of articles used for analyses was approved by all authors. Data extraction was split up between three authors. The following information was excerpted: author names and year of publication, types of participant groups, sample size, participant age in years (mean and standard deviation), mental age in years (mean and standard deviation), mean and standard deviation of IQ, secondary disorder, number of male and female participants, type of category under investigation (isolated vs. interrelated), language in which the study was carried out, task used to assess the central dependent variable including its type and the dependent measures derived from it, results (means and standard deviations, or, if these were not provided, alternative statistics). Risk of bias was considered in terms of diagnostic validation (similarly to Desaunay et al., 2020: validation via ADI-R and ADOS [regarded as low risk of bias] vs. validation via ADI-R or ADOS [regarded as medium risk of bias] vs. other [regarded as high risk of bias]). The coding scheme is available online (https://osf.io/gtj2p/). Data extraction and risk of bias ratings were double-checked by the second author. The included studies are summarized in Table 1.

**Table 1:** Characteristics of studies included in the meta-analysis

Study Name	Effect Size Identity Number (ID)	Type of Control Group	Autistic Group			Nonautistic Group		Risk of Bias	Type of Category	Type of Task	Type of Dependent Measure	Study Language	*g*
			*n* (Male)	Mean Age (*SD*)	Mean IQ	*n* (Male)	Mean Age (*SD*)						
Bott et al. (2006)	2; 1	Matched on IQ	12 (10)	30.00 (*SD* not reported, range 20-62)	Not reported	17 (6)	21.00 (*SD* not reported, range 19-45)	High	Isolated	Information-integration	Accuracy	English	−0.81; −0.37
Brown et al. (2010)	3	Matched on IQ	26 (24)	11.50 (1.20)	102.4	26 (24)	11.80 (1.60)	Medium	Isolated	Information-integration	Accuracy	English	0.39
Carmo et al. (2017)	4	Matched on IQ	21 (19)	27.24 (8.30)	Not reported	25 (22)	29.20 (10.89)	High	Interrelated	Rule-based	Response time	Portuguese	−1.33
Church et al. (2010)	5	Matched on IQ	20 (18)	9.40 (1.76)	107.93	20 (18)	9.50 (1.64)	Medium	Isolated	Prototype distortion	Accuracy	English	−1.13
Constable et al. (2018)	6; 7	Matched on IQ	23 (21)	40.35 (12.3)	111	24 (22)	38.71 (12.4)	Medium	Isolated	Rule-based	Accuracy	English	−0.64; −0.58;
Corbett et al. (2016)	8	Not matched on IQ	12 (7)	20.09 (2.66)	98.82	12 (5)	22.33 (2.42)	High	Isolated	Information-integration	Accuracy	Italian	−0.64
Daniels et al. (2018)	9	Not matched on IQ	16 (13)	12.13 (3.31)	102.75	17 (9)	11.53 (2.48)	High	Isolated	Information-integration	Accuracy	English	−0.12
Field et al. (2016)	11; 10	Matched on IQ	51 (45)	9.60 (3.35)	Not reported	45 (22)	4.63 (1.44)	Low	Interrelated	Rule-based	Accuracy	English	−0.28; 0.34
Froehlich (2008); Froehlich et al. (2012), Experiment 1^1^	15	Matched on IQ	24 (24)	25.17 (5.37)	108.58	25 (25)	24.76 (3.33)	Low	Interrelated	Prototype distortion	Accuracy	English	−0.36
Froehlich (2008/2012), Experiment 2 (Condition 1)	12	Matched on IQ	25 (25)	25.04 (5.22)	107.62	25 (25)	24.76 (3.33)	Low	Isolated	Rule-based	Accuracy	English	−0.92
Froehlich (2008/2012), Experiment 3 (Condition 1)	13	Matched on IQ	21 (21)	25.04 (5.22)	107.62	25 (25)	24.76 (3.33)	Low	Isolated	Information-integration	Accuracy	English	−0.6
Froehlich (2008/2012), Experiment 3 (Condition 2)	14	Matched on IQ	20 (20)	25.04 (5.22)	107.62	24 (24)	24.76 (3.33)	Low	Isolated	Rule-based	Accuracy	English	−0.22
Gastgeb et al. (2009)	16	Matched on IQ	51 (49)	18.40 (10.60)	105.8	49 (39)	19.10 (11.90)	Low	Isolated	Prototype distortion	Accuracy	English	−0.57
Gastgeb et al. (2011)	17	Matched on IQ	20 (20)	22.85 (6.16)	108.65	20 (20)	25.45 (6.29)	Low	Isolated	Prototype distortion	Accuracy	English	−0.92
Gastgeb et al. (2012)	18	Matched on IQ	20 (20)	23.40 (6.64)	110.7	19 (19)	26.42 (6.38)	Low	Interrelated	Prototype distortion	Accuracy	English	−0.85
Hartley & Allen (2014)	20; 19	Matched on IQ	17 (17)	9.70 (*SD* not reported, range 4.1-16.1)	Not reported	17 (6)	3.5 (*SD* not reported, range 2.2-6.4)	Low	Isolated	Rule-based	Accuracy	English	−1.34; −1.33
Hetzroni & Shalahevich (2018)	22; 21	Matched on IQ	34 (16)	9.40 (1.30)	Not reported	34 (33)	9.18 (0.90)	High	Interrelated	Rule-based	Accuracy	Hebrew	−1.38; −1.17
Hetzroni et al. (2019)	23	Matched on IQ	24 (18)	6.57 (0.87)	Not reported	24 (10)	5.63 (0.27)	High	Interrelated	Rule-based	Accuracy	Hebrew	−2.29
Hetzroni et al. (2019)	24	Not matched on IQ	24 (18)	6.57 (0.87)	Not reported	24 (15)	11.88 (2.29)	High	Interrelated	Rule-based	Accuracy	Hebrew	−1.08
Hoffmann & Prior (1982)	27; 28	Matched on IQ	10 (10)	11.25 (*SD* not reported, range 7.75-14.42)	87	10 (10)	8.75 (*SD* not reported, range 6.83-10.75)	High	Isolated	Rule-based	Accuracy	English	−2.26; −1.20
Hoffmann & Prior (1982)	26; 25	Not matched on IQ	10 (10)	11.25 (*SD* not reported, range 7.75-14.42)	87	10 (10)	11.25 (*SD* not reported, range 7.75-14.42)	High	Isolated	Rule-based	Accuracy	English	−2.82; −1.50
Kado et al. (2012)	30; 29	Matched on IQ	52 (39)	9.88 (2.42)	97.7	52 (41)	9.98 (2.79)	High	Isolated	Rule-based	Response time	Japanese	−0.50; −0.44
Kado et al. (2020): TD adolescent sample	31; 33	Not matched on IQ	39 (34)	12.24 (1.39)	96.9	39 (34)	12.26 (1.45)	High	Isolated	Rule-based	Response time	Japanese	−0.30; −0.00
Kado et al. (2020) ASD + ADHD adolescent sample	32; 34	Matched on IQ	39 (34)	12.24 (1.39)	96.9	21 (21)	11.81 (1.36)	High	Isolated	Rule-based	Response time	Japanese	−0.51; −0.36
Kado et al. (2020): TD child sample	37; 35	Not matched on IQ	30 (22)	8.22 (1.09)	94	30 (22)	8.18 (1.08)	High	Isolated	Rule-based	Response time	Japanese	−0.23; 0.13
Kado et al. (2020): ASD + ADHD child sample	38; 36	Matched on IQ	30 (22)	8.22 (1.09)	94	22 (17)	8.17 (1.23)	High	Isolated	Rule-based	Response time	Japanese	−0.80; 0.19
Kaland et al. (2008)	39	Matched on IQ	13 (13)	16.40 (2.84)	109	13 (13)	15.60 (3.07)	Low	Isolated	Rule-based	Response time	Danish	−0.38
Klinger & Dawson (2001)	40	Matched on IQ	12 (10)	14.42 (*SD* not reported, range 5.17-21.25)	Not reported	12 (10)	6.67 (*SD* not reported, range 4.33-11.67)	High	Isolated	Prototype distortion	Accuracy	English	−0.7
Klinger & Dawson (2001)	41	Matched on IQ	12 (10)	14.42 (*SD* not reported, range 5.17-21.25)	Not reported	12 (8)	14.17 (*SD* not reported, range 7.67 to 19.17)	High	Isolated	Prototype distortion	Accuracy	English	0.34
Mançe Çalişir et al. (2018)	42	Not matched on IQ	32 (17)	33.88 (9.38)	Not reported	23 (13)	27.39 (4.09)	High	Isolated	Rule-based	Response time	Turkish	−0.84
Mançe Çalişir et al. (2018)	43	Not matched on IQ	32 (17)	33.88 (9.38)	Not reported	17 (8)	24.59 (3.20)	High	Isolated	Rule-based	Response time	Turkish	−1.17
Maule et al. (2017)	46; 44; 45	Matched on IQ	16 (6)	24.60 (4.4)	105.5	16 (6)	24.50 (4.2)	High	Isolated	Information-integration	Accuracy	English	0.14; 0.22; 0.78
McGregor & Bean (2012) learner-driven condition	47	Matched on IQ	7 (6)	10.88 (2.10)	107	13 (7))	10.63 (2.12)	Medium	Isolated	Rule-based	Accuracy	English	−0.07
McGregor & Bean (2012) teacher-driven condition	48	Matched on IQ	15 (14)	11.05 (2.15)	110.46	12 (7)	10.47 (1.90)	Medium	Isolated	Rule-based	Accuracy	English	−1.09
Meyer (2014)	49; 50	Matched on IQ	18 (18)	11.15 (1.79)	114.22	16 (15)	10.53 (1.84)	Medium	Isolated	Prototype distortion	Accuracy	English	−.0.50; −0.07
Minshew et al. (1997)^2^	109	Matched on IQ	33 (29)	20.91 (9.69)	87.88	33 (29)	21.21 (9.99)	Low	Isolated	Rule-based	Accuracy	English	−0.21
Minshew et al. (2002)^3^	108; 106; 107	Matched on IQ	90 (*SD* not reported)	21.41 (9.68)	97.95	107 (*SD* not reported)	21.23 (9.81)	Low	Isolated	Rule-based	Accuracy	English	−0.40; −0.11; 0.56
Molesworth et al. (2005)	51	Matched on IQ	15 (15)	11.71 (1.65)	Not reported	15 (15)	11.73 (1.75)	High	Isolated	Prototype distortion	Accuracy	English	0.14
Molesworth et al. (2008)	52; 53	Matched on IQ	18 (16)	13.13 (2.02)	Not reported	18 (16)	12.88 (2.04)	High	Isolated	Prototype distortion	Accuracy	English	−0.50; −0.31
Molesworth et al. (2015)	54	Matched on IQ	15 (15)	13.14 (1.41)	99.27	17 (17)	13.16 (1.56)	Medium	Isolated	Prototype distortion	Accuracy	English	−0.34
Nader et al. (2022) feedback conditions	60; 59; 62; 61; 56; 58; 55; 57	Matched on IQ	26 (23)	9.40 (1.90)	103.25	28 (18)	9.90 (2.10)	Medium	Isolated	Information-integration	Accuracy (Effect Size IDs 55-58); response time (Effect Size IDs 59-62)	French	−5.18; −3.38; −3.02; −2.26; −0.86; −0.52; −0.22; 0.00
Nader et al. (2022) information conditions	63; 68; 66; 64; 65; 67; 70; 69	Matched on IQ	28 (24)	10.10 (2.00)	105.36	24 (18)	10.10 (1.40)	Medium	Isolated	Information-integration	Accuracy (Effect Size IDs 63-66); response time (Effect Size IDs 67-70)	French	−0.73; −0.59; −0.24; −0.14: 0.12 0.13; 1.64; 1.94
Naigles et al. (2013) Experiment 1	71	Matched on IQ	26 (24)	12.77 (2.41)	108.4	23 (20)	12.83 (1.57)	Medium	Interrelated	Information-integration	Accuracy	English	−0.7
Naigles et al. (2013) Experiment 2	72	Matched on IQ	12 (7)	11.33 (2.18)	90.64	11 (8)	10.17 (1.46)	Low	Interrelated	Information-integration	Accuracy	English	−0.68
Park et al. (2014)	73	Not matched on IQ	75 (*SD* not reported)	7.91 (4.22)	87.99	77 (*SD* not reported)	8.14 (5.17)	Low	Isolated	Rule-based	Response time	Korean	−0.47
Plaisted et al. (1998)	74	Not matched on IQ	8 (*SD* not reported)	28.75 (not reported)	Not reported	10 (*SD* not reported)	28.5 (*SD* not reported)	High	Interrelated	Information-integration	Accuracy	English	−1.09
Potrzeba et al. (2015)	82; 80; 76; 81; 78; 75; 79; 77	Not matched on IQ	32 (27)	2.73 (0.45)	Not reported	35 (29)	1.69 (0.13)	High	Isolated	Rule-based	Accuracy	English	−0.58; −0.36; −0.35; −0.26 0.00; 0.20; 0.44; 0.54
Powell (2016); Powell et al. (2017)^3^ Woodcock-Johnson Concept Formation Test	83	Matched on IQ	28 (24)	49.00 (11.70)	113.2	30 (23)	48.70 (12.10)	Medium	Isolated	Rule-based	Accuracy	English	−0.63
Powell (2016/2017) Prototype effect	84	Matched on IQ	29 (24)	49.00 (11.70)	113.2	30 (23)	48.70 (12.10)	Medium	Isolated	Prototype distortion	Accuracy	English	−0.41
Rumsey (1985)	85	Matched on IQ	9 (9)	27.00 (7.00)	104	10 (10)	28.00 (5.00)	High	Isolated	Rule-based	Response time	English	−0.89
Sapey-Triomphe et al. (2018)	89; 87; 86; 88	Matched on IQ	20 (15)	33.60 (10.00)	113	20 (16)	30.80 (6.90)	Medium	Isolated	Rule-based	Accuracy	French	−1.08; −0.92; −0.18; −0.04;
Schipul & Just (2016)	90	Matched on IQ	16 (14)	26.50 (6.40)	113.8	16 (14)	25.40 (7.70)	Low	Isolated	Prototype distortion	Response time	English	−0.71
Schipul et al. (2012)	94; 91; 93; 92	Matched on IQ	18 (16)	22.40 (9.60)	106.2	18 (17)	22.40 (4.50)	Low	Isolated	Information-integration	Accuracy (Effect Size ID 91); response time (Effect Size IDs 92-94)	English	−0.20; −0.10; −0.01; 0.33
Shu et al. (2001)	95	Not matched on IQ	26 (*SD* not reported)	aged 6 to 12	80	52 (not reported)	matched for age	High	Isolated	Rule-based	Response time	Standard Chinese	−1.09
Solomon et al. (2011)	96; 98; 97	Not matched on IQ	20 (19)	9.71 (1.17)	Not reported	22 (21)	10.25 (1.43)	Medium	Isolated	Rule-based	Accuracy	Hebrew	−1.08; −0.98; −0.90
Soulières et al. (2011)	99	Matched on IQ	16 (12)	17.80 (*SD* not reported, range 11-29)	108.3	16 (12)	16.70 (*SD* not reported, range 11-27)	Low	Isolated	Rule-based	Accuracy	French	−1.05
Tecoulesco et al. (2021)	100	Not matched on IQ	16 (14)	6.50 (0.53)	85.3	30 (25)	5.60 (0.35)	Medium	Interrelated	Rule-based	Accuracy	English	−0.6
Tovar et al. (2020)	101	Matched on IQ	24 (21)	8.29 (2.46)	Not reported	24 (21)	4.49 (0.54)	High	Isolated	Rule-based	Accuracy	Spanish	−0.4
Vladusich et al. (2010) Experiment 1	103; 102	Matched on IQ	19 (19)	18.40 (2.95)	106.5	21 (19)	21.00 (2.70)	Low	Interrelated	Prototype distortion	Accuracy (Effect Size ID 102); response time (Effect Size ID 103)	English	−1.20; −0.86
Vladusich et al. (2010) Experiment 2	104; 105	Matched on IQ	13 (13)	19.20 (3.30)	113.1	18 (18)	19.10 (2.10)	Low	Interrelated	Rule-based	Accuracy (Effect Size ID 104); response time (Effect Size ID 105)	English	−0.54; −0.03
Williams et al. (2014)^3^	110	Matched on IQ	65 (*SD* not reported)	18.83	Not reported	65 (*SD* not reported)	19.17	Low	Isolated	Rule-based	Accuracy	English	−0.19
Williams et al. (2015)	111	Matched on IQ	30 (25)	29.10 (10.90)	104.7	57 (52)	28.80 (9.45)	Low	Isolated	Rule-based	Accuracy	English	−0.48
Wright et al. (2016)	112	Matched on IQ	18 (15)	6.61 (1.29)	102.82	18 (15)	6.41 (1.75)	Medium	Isolated	Rule-based	Accuracy	unclear	0.6

^1^Since Froehlich (2008) and Froehlich et al. (2012) worked with the same sample (except for two participants from the autistic group, who were included in the analyses of Froehlich, 2008, but excluded from the analyses in Froehlich et al., 2012), and Froehlich et al. (2012) reported a study also presented in Froehlich (2008; here named “Experiment 1”), we treated the data of both records as dependent from each other and included the study reported twice only once.

^2^Williams et al. (2014) stated that their sample overlapped with the samples of Minshew et al. (1997, 2002). Thus, the samples of these studies were modelled as statistically dependent within our multi-level random effects model.

^3^Since Powell (2016) and Powell et al. (2017) worked with the same sample and the *Woodcock-Johnson Concept Formation Test* was reported in both records, we treated the data of both works as dependent from each other and included results of the *Woodcock-Johnson Concept Formation Test* only once.

Several *g* values per row reflect group comparisons regarding multiple dependent measures, which were accounted for in the multilevel meta-analysis*.*

### Coding of moderator variables

Age of autistic participants was coded as the group mean of chronological age in years and implemented as continuous moderator. Similarly, IQ of autistic participants mirrored the continuous group mean of intelligence in units of the IQ scale. Year of publication was incorporated as another continuous predictor in units of whole years. Risk of bias was included as categorical moderator; levels were low, medium, and high (see data extraction). Type of control group was employed as categorical moderator distinguishing between nonautistic groups matched vs. not matched on IQ. A group was deemed to be matched if the original study reported it as such. If only mental age—but not IQ—was reported, we utilized mental age as a proxy of IQ. This means that we considered groups to be matched on IQ if they were reported to be matched on mental age. However, studies reporting only the mental age but not the IQ of their participants were excluded from the moderator analysis on IQ. Type of task was incorporated as a categorical moderator with levels being information-integration, prototype distortion, and rule-based. The strategy leading to optimal performance was easy to verbalize in rule-based tasks and hard or impossible to verbalize in information-integration tasks. Prototype distortion tasks manifested themselves in the way the stimulus material was constructed, namely through first establishing a prototype and subsequently creating random distortions of the prototype. Type of dependent measure was employed as a categorical moderator comparing accuracy with response time. Measures were considered to represent accuracy if the correctness of responses was at their core, whereas measures coded as response time were about the speed with which a response was achieved. Study language was coded as a categorical moderator. Within moderator analyses, a language was included only if it occurred across a minimum of five effect sizes. In line with this, languages covered by less than five effect sizes were excluded from the moderator analysis on study language. Percentage of male participants was modelled as continuous moderator reflecting the proportion of males within autistic groups.

**Fig. 1:**
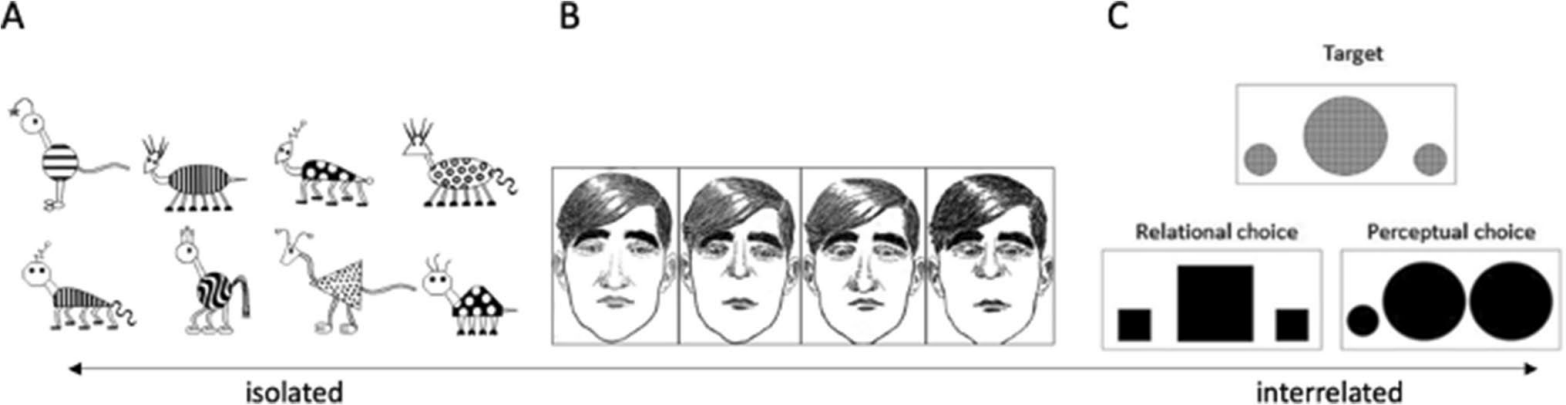
Examples of the distinction between isolated and interrelated categories. *Note. *A = Category is based on one feature: “Rogs” have lines and “Zips” have dots (Froehlich, 2008; with kind permission of Alyson Froehlich). B = Category is based on features that can be regarded in isolation: face length, nose length, nose width, distance between eyes (Gastgeb et al., 2009; with kind permission of John Wiley and Sons). C = Category is determined by a relation; perceptual similarities like shape are not diagnostic (Hetzroni & Shalahevich, 2018; with kind permission of Springer Nature)

Type of category was considered in terms of the isolated vs. interrelated distinction (Goldstone, 1996; see Introduction). A category was deemed isolated when looking at one or several features in isolation was sufficient to determine membership (e.g., Froehlich, 2008, Experiment 3). In contrast, when classification necessitated looking at relations between stimulus components, a category was considered interrelated (e.g., Hetzroni & Shalahevich, 2018). In line with the view that the isolated-interrelation distinction is continuous rather than categorical, there were ambiguous cases. Here, we checked whether the majority of relevant features could be regarded in isolation—in that case, the category was termed isolated—or required the inclusion of relations with other features to arrive at correct categorization—this presented an instance of interrelated categories. For example, the face stimuli used by Gastgeb et al. (2009) varied in terms of face length, nose length, nose width, and distance between eyes. Since only distance between eyes is a relational feature and the remaining face properties could be considered in isolation, we deemed this study to investigate isolated categories. See Fig. 1 for an illustration of the distinction between isolated and interrelated categories.

### Statistical analysis

Effect sizes were calculated in terms of the standardized mean difference, as Hedges’ *g*, using formulas provided in Borenstein (2009) and Lipsey and Wilson (2001), Comprehensive Meta-Analysis software (Biostat Inc.), the online calculator provided by the Campbell Collaboration (https://www.campbellcollaboration.org/escalc/html/EffectSizeCalculator-SMD4.php), and the esc package in R (R Version 4.0.3). All further meta-analytical steps were carried out using the R metafor package (Viechtbauer, 2010). Meta-analytical code can be accessed online (https://osf.io/gtj2p/).

Effect sizes were weighted by their inverse variance. Negative effect sizes indicated lower-level performance of the autistic group compared with the nonautistic group. Imprecision was estimated through 95% confidence intervals. For each of the effect sizes, we checked data for dependency. Two types of statistical dependency were identified: First, the same participants were included in more than one pairwise group comparison, and second, more than one dependent measure was assessed in the same participants. To account for dependency, we implemented a random effects multilevel meta-analysis (Hox et al., 2002). Individual effect sizes constituted Level 1; these were modelled to be nested within dependent comparisons, which represented Level 2. Dependent comparisons in turn were considered to be nested within independent studies constituting Level 3. We determined heterogeneity by means of χ^2^ (*Q*) and Higgins *I*^*2*^ tests. An influence analysis was conducted based on hat values and Cook’s distance (Belsley et al., 1980; Cook, 1977). Potential moderators in terms of risk of bias, type of control group, type of category, study language, type of task, type of dependent measure, age of autistic group, IQ of autistic group, year of publication, and percentage of male participants within autistic group were investigated using a series of meta-regression models. Publication bias was assessed through a funnel plot and Egger’s test (Egger et al., 1997; Light & Pillemer, 1984). We adopted the standard 5% significance level for all inferential tests.

## Results

### Number of studies

**Fig. 2:**
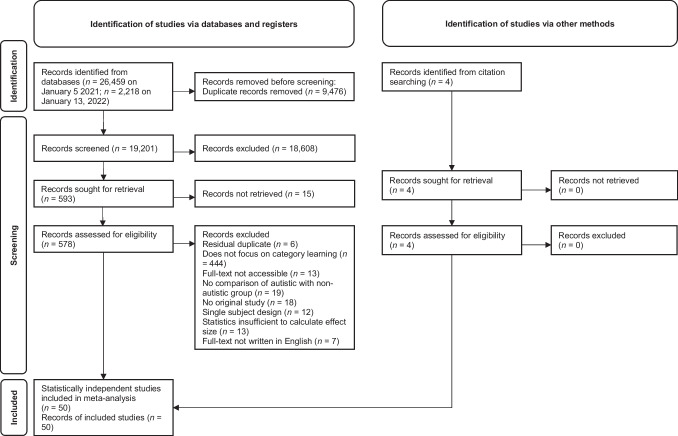
PRISMA flowchart of the literature search

Our literature search detected 26,459 database entries on January 5, 2021, and 2,218 additional documents published between January 5, 2021 and January 13, 2022 (see Fig. 2). After removing duplicates, 19,201 records were left for further inspection. Screening of titles and abstracts led to the exclusion of 18,608 documents that did not meet inclusion criteria, and a further 15 records for which abstracts were not accessible. Full texts were sought for the remaining 578 database hits. Out of these documents, six were excluded since they were residual duplicates; 444 were excluded because they did not deal with the acquisition of categories/prototypes/concepts/schemata; 13 records were excluded since full texts were not available; 19 were excluded because they did not involve a group comparison of autistic individuals with controls; 18 titles were excluded because they did not report original research; 12 were excluded because they reported a single-subject design; 13 were excluded due to missing statistics; and seven records were excluded since the full text was not written in English. In addition, four titles were identified via manual search/search of reference lists. Overall, 50 records, which provided 50 statistically independent comparisons and 112 effect sizes, were included in our meta-analysis.

### Characteristics of studies and samples

The current pool of studies involved 1,220 autistic and 1,445 nonautistic participants (for a study overview, see Table 1). For individual studies, sample sizes of autistic groups varied between *n* = 7 (learner-driven condition; McGregor & Bean, 2012) and *n* = 90 (Minshew et al., 2002), interquartile range (*IQR*): [16, 27.5], whilst those of nonautistic groups varied between *n* = 10 (Hoffmann & Prior, 1982) and *n* = 107 (Minshew et al., 2002), *IQR*: [16, 29.5]. Mean chronological ages of autistic groups ranged from 2.73 years (Potrzeba et al., 2015) to 49.00 years (Powell, 2016/2017), *IQR*: [9.70, 23.26], whereas those of nonautistic groups ranged from 1.69 years (Potrzeba et al., 2015) to 48.70 years (Powell, 2016/2017), *IQR*: [10.10, 24.50]. Regarding participant gender, percentage of male volunteers in autistic groups varied between 37.50% (Maule et al., 2017) and 100.00% (for instance, Froehlich, 2008; Froehlich et al., 2012; Gastgeb et al., 2011, 2012; Hartley & Allen, 2014; Kaland et al., 2008; Meyer, 2014; Molesworth et al., 2015; Rumsey, 1985; Vladusich et al., 2010), *IQR*: [83.33%, 96.15%]; percentage of male respondents in nonautistic groups ranged from 35.29% (Bott et al., 2006) to 100.00% (for instance, Froehlich, 2008; Froehlich et al., 2012; Gastgeb et al., 2011, 2012; Kado et al., 2020; Kaland et al., 2008; Molesworth et al., 2015; Rumsey, 1985; Vladusich et al., 2010), *IQR*: [73.30%, 95.20%]. For 75% of comparisons, participant groups were matched on IQ, leaving 25% of comparisons for which groups were not matched on IQ. IQ in autistic groups ranged from 80.00 (Shu et al., 2001) to 114.22 (Meyer, 2014), *IQR*: [99.16, 108.74]. In terms of the type of categories, 80% of studies addressed isolated categories, whereas only 20% examined interrelated categories. Looking at the type of tasks, 28.57% of group comparisons concerned information-integration tasks, 15.18% addressed prototype distortion tasks, and 56.25% referred to rule-based tasks. Table 2 gives an overview of the tasks used within the seven most influential studies based on the nine most highly weighted effect sizes. Risk of bias was low in 30% of studies, medium in a further 30% of studies, and high in 40% of studies. Category learning as dependent measure was indexed by either accuracy (72% of group comparisons) or response time (28% of group comparisons). Accuracy was typically utilized as absolute (e.g., Powell, 2016) or relative (e.g., Sapey-Triomphe et al., 2018) number of correct responses, or defined ex negativo as number of errors (e.g., Williams et al., 2015). Response time was reflected by the number of trials needed to achieve a certain accuracy criterion (e.g., Schipul & Just, 2016; Shu et al., 2001) or by the time needed to respond to a single stimulus (e.g., Nader et al., 2022). Confined to languages for which there were at least five effect sizes, 64% of studies were conducted in English, 6% in Hebrew, 6% in Japanese, and 8% in French.

**Table 2:** Overview of the tasks used within the seven most influential studies based on the nine most highly weighted effect sizes

Study	Task Name	Task Description	Type of Feedback Provided
Park et al. (2014) (also used in Kaland et al., 2008; Mançe Çalişir et al., 2018; Rumsey, 1985; Shu et al., 2001)	Wisconsin Card Sorting Test (WCST)	Four stimulus cards are presented that vary in color, shape, and number. In each trial, participants need to match an additional card with one of the four stimulus cards. Feedback informs as to whether the decision was correct or incorrect. The correct sorting dimension needs to be inferred based on feedback.	Feedback after each response indicates response accuracy (correct vs. incorrect)
Potrzeba et al. (2015)	Shape bias task	Participants view triplets of objects, one of which is the target, one matches the shape of the target (shape match), and one matches the color of the target (color match). Two types of trials are implemented: name trials during which the target object is named (e.g., “dax”); and no-name trials during which the target object is not named. Intermodal preferential looking times (i.e., dwell times of the shape matches) serve as dependent measure.	None provided
Hetzroni & Shalahevich (2018)	Matching-to-sample categorization task	In each trial, participants are presented with a set of three three-object stimuli (black-and-white geometric shapes) consisting of one standard stimulus and two target stimuli. In condition 1, one of the target stimuli is structurally related to the standard stimulus and the other one is unrelated to the standard stimulus. In condition 2, one of the target stimuli is structurally related to the standard stimulus and the other one is basically-perceptually related to the standard stimulus. Participants are asked to decide which of the two target stimuli is most closely related to the standard stimulus.	None provided
Gastgeb et al. (2009)	Face prototype task	Participants view 14 line-drawn faces varying along four features (face length, nose length, nose width, interocular distance). Faces are presented one by one for 2 seconds each. Non-average manifestations of each facial feature are seen 12 times, whereas average instantiations are seen only twice. Immediately after this familiarization phase, volunteers participate in a forced-choice test with two novel faces side by side: One of these is the numerical average of the varied facial features (i.e., the mean prototype). The other face is the mode of those facial attributes (i.e., the mode prototype). Participants are asked to decide which of the faces looks more familiar to them.	None provided
Minshew et al. (2002)	Halstead Category Test	Stimuli (mainly geometric shapes) are presented together with four numbered keys. Participants are instructed that the stimuli in each of the seven subtests share some principle or idea represented by one of the key numbers. Auditory feedback is given as to whether the button press was correct or wrong. Participants are to identify the underlying principle/idea based on that feedback.	Feedback after each response indicates response accuracy (correct vs. incorrect)
Minshew et al. (2002)	Goldstein-Scheerer Object Sorting Test	Test stimuli consist of 30 familiar objects that can be categorized according to several principles (e.g., function, material, color). In the first part, participants are required to find objects matching the category that an object presented by the examiner belongs to. Next, participants are asked to piece together items that belong together and to provide a verbal rationale. Finally, participants are required to shift categories or regroup the same objects in another way.	None provided
Kado et al. (2012) (also used in Kado et al., 2020)	Keio Wisconsin Card Sorting Test (Keio WCST)	WCST version with the following modifications of the original test: Fewer cards need to be sorted; the order of the response cards is changed; the cards need to be sorted twice - between the two rounds (called “steps”), participants receive an instruction.	Feedback after each response indicates response accuracy (correct vs. incorrect)

Finally, the full data set was available from the authors upon request in one case only (i.e., Tovar et al., 2020) and not available for the remaining studies.

### Meta-analysis

Our primary research question addressed whether and to what extent autistic individuals differ in category learning from nonautistic individuals. According to meta-analysis of the 112 effect sizes from 50 records, autistic individuals showed lower-level performance in category learning compared with nonautistic individuals. This effect was medium-sized and statistically significant, *g* = −0.55, 95% CI [−0.73, −0.38], *p* < .0001 (Table 3, supplementary Fig. 1, see https://osf.io/gtj2p/). Presence of heterogeneity was indicated by a significant *Q* statistic, *Q*(111) = 617.88, *p* < .0001, which is addressed in the following section (moderator analyses). Furthermore, total *I*^2^ was 85.14% indicating a substantial amount of true variance (vs. sampling error) in effect size estimates, the majority of which came from the within-study cluster, *I*^2^_*Level2*_ = 55.37%, compared with the between-study cluster, *I*^2^_*Level3*_ = 29.77%. Together, these results suggest that autism is associated with medium-sized lower-level category learning skills and that the effect sizes differ systematically between studies due to factors varying within-study (e.g., type of control group, type of dependent measure). Robustness of the effect was confirmed across influences analyses: Hat values ranged from .0031 to .0160, thus all scores were below the critical cut-off of 3/*k* = 3/112 = .0268 (Harrer, 2022). Similarly, Cook’s distance varied between .0000 and .0388, meaning that scores were below the threshold of .45 (Harrer, 2022) that would signal an influential study.

**Table 3:** Meta-analytic results

		Number of Studies	Number of ES	Effect Size Estimate					*p* informs whether …	*Q*
				Parameter	95% *CI*	*SE*	*t*	*p*		
Overall Estimate		50	112	*g* = −0.55	[−0.73, −0.38]	0.09	−6.18	< .0001	… *g* is different from 0	617.88***
Categorical Moderators										
Risk of bias	High (intercept)	20	45	*g* = −0.60	[−0.88, −0.32]	0.14	−4.18	<.0001	… *g* is different from 0	610.39***
	Medium (vs. high)	15	37	*g* = −0.54	[−0.98, −0.11]	0.22	0.27	.787	… effect of moderator is different from 0	
	Low (vs. high)	15	30	*g* = −0.51	[−0.95, −0.07]	0.22	0.41	.684	… effect of moderator is different from 0	
Type of control group	Matched on IQ (intercept)	36	84	*g* = −0.53	[−0.73, −0.33]	0.10	−5.20	< .0001	… *g* is different from 0	616.11***
	Not matched on IQ (vs. matched on IQ)	14	28	*g* = −0.64	[−1.02, −0.26]	0.19	−0.60	.551	… effect of moderator is different from 0	
Type of category	Isolated (intercept)	40	95	*g* = −0.50	[−0.69, −0.30]	0.10	−5.09	<.0001	… *g* is different from 0	593.56***
	Interrelated (vs. isolated)	10	17	*g* = −0.81	[−1.26, −0.37]	0.22	−1.44	.154	… effect of moderator is different from 0	
Study language	English (intercept)	32	65	*g* = −0.46	[−0.69, −0.24]	0.11	−4.04	< .0001	… *g* is different from 0	517.43***
	Hebrew (vs. English)	3	7	*g* = −1.28	[−1.98, −0.57]	0.36	−2.30	.023	… effect of moderator is different from 0	
	Japanese (vs. English)	3	10	*g* = −0.29	[−0.94, 0.35]	0.33	0.52	.607	… effect of moderator is different from 0	
	French (vs. English)	4	21	*g* = −0.70	[−1.26, −0.14]	0.28	−0.85	.398	… effect of moderator is different from 0	
Type of task	Information-integration (intercept)	11	32	*g* = −0.41	[−0.77, −0.04]	0.18	−2.21	.029	… *g* is different from 0	611.03***
	Prototype distortion (vs. information-integration)	12	17	*g* = −0.61	[−1.14, −0.09]	0.26	−0.77	.441	… effect of moderator is different from 0	
	Rule-based (vs. information-integration)	17	63	*g* = −0.60	[−1.03, −0.17]	0.22	−0.89	.376	… effect of moderator is different from 0	
Type of dependent measure	Accuracy (intercept)	36	81	*g* = −0.49	[−0.69, −0.29]	0.10	−4.83	<.0001	… *g* is different from 0	613.89***
	Response time (vs. accuracy)	14	31	*g* = −0.74	[−1.09, −0.40]	0.17	−1.47	.144	… effect of moderator is different from 0	
Continuous Moderators										
Age of autistic group		50	112	*b* = −0.00	[−0.02, 0.02]	0.01	−0.05	.957	… effect of moderator is different from 0	616.66***
IQ of autistic group		36	81	*b* = 0.012	[−.0.02, 0.04]	0.01	0.87	.386	… effect of moderator is different from 0	470.23***
Year of publication		50	112	*b* = 0.01	[−0.01, 0.04]	0.01	1.26	.211	… effect of moderator is different from 0	617.86***
Percentage of male participants in autistic group		46	105	*b* = −0.00	[−0.02, 0.01]	0.01	−0.66	.512	… effect of moderator is different from 0	557.91***

ES = effect sizes

### Moderator analyses

**Fig. 3:**
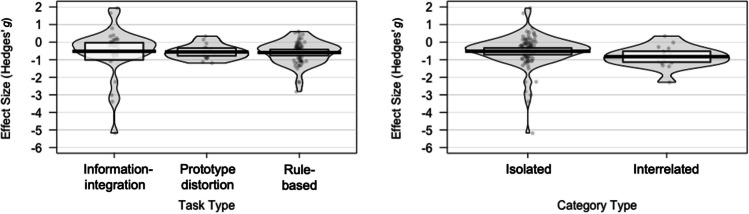
Visualized effects of types of task and category. *Note.* Pirate plots reflecting effect size by task and category types, showing raw data points, a horizontal line reflecting the mean, a rectangle representing the 95% confidence interval, and a bean representing a smoothed density

A series of moderator analyses checked whether the significant amount of between-study heterogeneity can be explained by two types of factors: Firstly, categorical variables including risk of bias (low vs. medium vs. high), type of control group (matched vs. not matched on IQ), type of category (isolated vs. interrelated; see Fig. 3), study language (English vs. Hebrew vs. Japanese vs. French), type of task (information-integration vs. prototype distortion vs. rule-based; see Fig. 3), type of dependent measure (accuracy vs. response time); and secondly, continuous variables, namely age of autistic group, IQ of autistic group, year of publication, and percentage of male participants within autistic group. Table 3 provides an overview of the overall meta-analytic effect as well as the effect of these moderator variables. For categorical moderators, the level presented first serves as the intercept. In this case, the *p*-value of the corresponding effect size parameter indicates whether the effect of the intercept differs significantly from zero. The effect of each of the subsequent moderator levels is compared to that of the intercept, so that the *p* values of these effects reflect whether the effect of the moderator level differs significantly from that of the intercept, and hence suggest whether the respective variable exerts a moderating influence. For continuous moderators, the effect size parameter is a regression weight, *b,* so that *p* values here indicate whether there is moderation by way of a significant linear relationship between moderator and outcome. As can be seen from Table 3, only one of the variables under investigation was found to be a significant moderator, namely study language. In particular, studies conducted in Hebrew were associated with a more negative effect than studies conducted in English (*g* = −1.28 vs. *g* = −0.46, *p* = .023). In contrast, none of the remaining variables moderated the overall effect (*p*s > .13).

### Publication bias

**Fig. 4:**
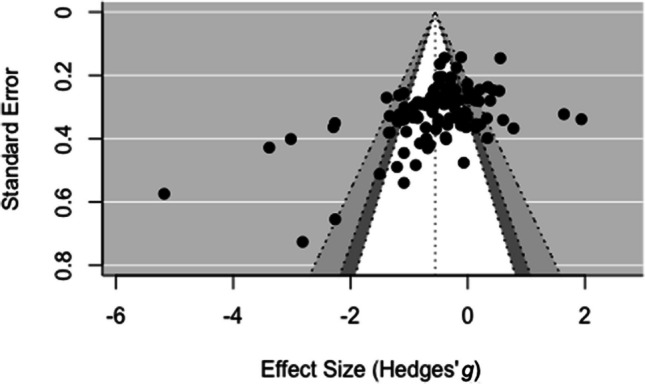
Funnel plot of the results. *Note*. Effect sizes (in units of Hedges’ *g*) on the *x*-axis are plotted against their standard error on the *y*-axis. The dotted vertical line represents the mean meta-analytical effect. Within the funnel shape, the white area reflects the 90% CI of the mean effect, the dark-grey area reflects the 95% CI of the mean effect, and the light grey area reflects the 99% CI of the mean effect

The current pool of studies included published as well as unpublished work, for example, doctoral dissertations. According to the tests we carried out, presence of publication bias could not be ruled out. Visual inspection of the funnel plot revealed a substantial degree of asymmetry as there were more data points from relatively imprecise studies to the left than to the right of the mean effect (see Fig. 4). That is, studies with smaller samples and concomitant larger standard errors reported greater negative effects than studies with larger samples and concomitant smaller standard errors. Resonating with this, the slope of Egger’s regression test for funnel plot asymmetry was significantly negative, *b* = −6.41, *SE* = 1.16, *t*(110) = −5.51, *p* < .0001, indicating that the precision of the measured effect was significantly linked with the magnitude of the effect. As opposed to this, if there is no publication bias, a symmetric distribution of data points around the mean effect alongside a nonsignificant Egger’s test is to be expected (Egger et al., 1997).

## Discussion

In the present article, we aimed to obtain a comprehensive research overview of category learning in autistic persons. Firstly, we investigated whether and to what extent autistic individuals differ in category learning from nonautistic individuals. Based on earlier narrative reviews (Mercado et al., 2020; Patry & Horn, 2019; Vanpaemel & Bayer, 2021), we predicted lower performance levels for autistic compared with nonautistic persons. This hypothesis was supported: Within our meta-analysis, results of a multilevel random effects model indicated that overall autistic individuals have lower-level skills of category acquisition compared with nonautistic individuals. This total effect was of medium size (*g* = −0.55) and statistically significant. Both accuracy (*g* = −0.49) and response time (*g* = −0.74) in categorization were affected. In sum, this is the first quantitative synthesis to evidence differences in category learning for autistic individuals. These differences suggest a form of atypical category learning leading to difficulties in fluency with regard to correctness and speed of categorization (e.g., Jimenez et al., 2021).

Second, resonating with earlier narrative review articles (e.g., Dovgopoloy & Mercado, 2013), there was a significant amount of heterogeneity between the effect sizes included in our meta-analysis. Therefore, we examined whether moderator variables would account for the observed heterogeneity. This turned out to be the case for only one of the moderator variables under examination, that is, study language—studies conducted in Hebrew yielded more negative effects than studies carried out in English (*g* = −1.28 vs. *g* = −0.46). Since no direct comparison of category acquisition in Hebrew and English has been carried out in the extant literature, this effect is difficult to interpret. There is also a potential confound of study language with type of category. In particular, 18.46% of the effect sizes linked with English studies addressed relational categories, whereas this was the case for 57.14% of the effect sizes linked with Hebrew studies. Beyond this, the number of studies in Hebrew was very low (*k* = 3). In sum, this pattern should be treated with great caution and points out a need for further research in this area. For the remaining moderator variables, namely age, year of publication, risk of bias (low vs. medium vs. high), type of control group (matched vs. not matched on IQ), IQ of autistic group, percentage of male autistic participants, type of category (isolated vs. interrelated), type of task (information-integration vs. prototype distortion vs. rule-based), and type of dependent measure (accuracy vs. response time), meta-regression models did not detect statistically significant effects. Thus, although we considered a large number of variables based on existing literature, we were not able to explain the variability observed in a fully reliable manner.

The overall quality of original studies was mixed. Risk of bias estimates related to validation of the autism diagnosis were very variable, ranging from low to high with approximate equal distribution. Further, to ascertain whether differences between autistic and nonautistic groups are specifically related to autism, it is important to closely match participant groups on all relevant characteristics except for presence of autism. In order that differences in general cognitive potential can be ruled out as explanatory factors, studies within autism research typically match participant groups on IQ (see Jarrold & Brock, 2004; Mottron, 2004). In the current pool of studies, the majority of investigations (i.e., 75%) did rely on participants’ IQ for group matching.

Another factor related to study quality is sample size, as small samples are characterized by instability of the mean estimates they provide (see Bishop et al., 2022; Tversky & Kahneman, 1971). In meta-analyses, this sampling error is acknowledged through study weight. Nevertheless, this weight is relative to the corpus of included studies, and so cannot compensate if sample sizes are low overall. Assuming a two-group design, total sample size of the currently included studies had a median of *N* = 41. Post hoc power analysis revealed that this sample size has a power of 88% to detect a medium-sized effect of *d* = 0.5 in a two-tailed *t* test for paired samples at the standard 5% significance level, and a power of 47% to identify a small-sized effect of *d* = 0.3 in the same sort of inference test. Thus, the present evidence base did not have sufficient power for detecting small effects, hence may be marked by a certain degree of imprecision. Still, low sample sizes are typical of autism research (Tager-Flusberg, 2004), so this limitation is by no means specific to the current meta-analysis. Furthermore, those indicators of study quality that were amenable to and thus included in moderator analysis, namely risk of bias and type of control group [matched vs. not matched on IQ], were not shown to impact on the results. This means that studies with lower quality did not seem to produce results different from those higher in quality.

### Strengths and limitations

In this article, we presented the first-ever meta-analysis of category learning in autistic individuals. As distinct from earlier reviews, our synthesis aimed to incorporate all kinds of mental representations and all types of categories, based on preceding decades of research into human categorization. In addition, it provides a quantitative summary and used statistical methods in order to explain heterogeneity between studies. Related to this all-encompassing approach is the relatively large number of original studies/effect sizes included. Carter et al. (2019) demonstrated that meta-analytical methods involving 60 studies have excellent power. The present number of investigations—50 studies reporting 112 effect sizes—comes comparatively close to this figure. We also used an up-to-date statistical method, that is, a multilevel approach, to account for dependencies between effect sizes and avoid an overestimation of effects by erroneously assuming independence.

Nonetheless, there is reason to assume that the true difference in category learning between autistic and nonautistic individuals is somewhat lower than what is suggested by the total effect, *g* = −0.55. In particular, inspection of the funnel plot and Egger’s test revealed an asymmetric distribution of effect sizes in the sense that effects suggesting lower-level performance of autistic individuals were overrepresented. As this asymmetry could trace back to publication bias, future work could produce a more balanced picture if researchers and publication outlets tried to publish findings irrespective of statistical significance and direction of effects. Another limitation is linked with study heterogeneousness. Even though checks in terms of hat values and Cook’s distance suggested that our findings were robust, so not biased by individual influential studies, effect sizes were still afflicted with a significant amount of heterogeneity; and this heterogeneity could not be fully explained by any of our moderator variables. This means that currently unknown moderator variables could account for the heterogeneity. Further work is needed to clarify this issue.

As applicable to many lines of autism research, the present meta-analysis is limited in establishing causal links, here between the presence of autism and category learning skills. This is because, firstly, groups of participants were self-selected. Secondly, although the majority of studies, that is, 75%, matched participant groups on IQ, and many of them also on age and gender, whether these are the only variables critical to category learning is unknown. Thirdly, considering the relatively low sample sizes, it is not guaranteed that these further variables were randomly distributed across participant groups. In sum, the presence of autism is possibly not the only difference between participant groups that is apt to explain variations in category learning.

Given that we reported the first comprehensive meta-analysis on this topic, it is difficult to draw straightforward comparisons with previous syntheses. Still, it is interesting to see whether the present work arrived at similar conclusions as earlier reviews. In their article, Patry and Horn (2019) reported small- to large-sized disadvantages for prototype formation, and mostly medium- to large-sized disadvantages for both categorization and schema development. The medium-sized total effect obtained in our meta-analysis generally suggested lower differences between autistic and nonautistic individuals, which was probably still overstated due to publication bias. Hence, Patry and Horn’s (2019) synthesis is likely to be subject to even greater bias.

Mercado et al. (2020) emphasized that findings on learning *perceptual* categories in autistic individuals are heterogeneous, whilst they considered the bulk of the evidence to gravitate toward dysfunctional category learning. On the one hand, the findings of the present meta-analysis specify this view to the extent that they quantify effects and corresponding heterogeneity. On the other hand, they extend Mercado et al.’s (2020) view as they demonstrate that the effect goes beyond learning perceptual categories and seems to apply to all category types under investigation.

Finally, Vanpaemel and Bayer’s (2021) conclusions about prototype-based category learning in autism basically resonate with Patry and Horn’s (2019) inferences on prototype formation. In an attempt to explain divergent findings, Vanpaemel and Bayer (2021) focused on task characteristics. They hypothesized that tasks suggesting a prototype-based mental representation would pose greater challenges for autistic individuals than tasks prompting an exemplar-based mental representation. Since the vast majority of studies did not provide evidence about the type of mental representation utilized or built up by participants, we were not able to formally test this assumption within moderator analysis. However, such a test seems to be a promising objective for future work.

To what population and what outcomes do the present results generalize? Most of the included studies worked with older children, adolescents, or young adults, so inferences about these age groups are feasible in principle; in contrast, younger children and older adults were clearly underrepresented. Regarding gender, the majority of study samples involved approximately 83 to 96% male participants. Zeidan et al. (2022) reported a male-to-female ratio of 4.2 in autism corresponding to roughly 81% males among autistic persons. Therefore, male participants were slightly overrepresented in the current meta-analysis. Furthermore, most studies relied on verbal materials requiring at least basic language skills. Thus, it can be assumed that those studies worked with high-functioning autistic individuals who are not representative of the entire autism spectrum. In support of this, the average IQ of the autistic groups in most cases (referring to the interquartile range) ranged between 99.16 and 108.74. This bias in selection might go back to the frequently observed strategy that groups of autistic and typically developing groups are matched on IQ. Although autistic individuals with low levels of intelligence are therefore neglected, recent research demonstrates that this group of individuals may constitute a smaller portion of all autistic persons than thought previously (Billeiter & Froiland, 2023; Katusic et al., 2021; Wolff et al., 2022).

Looking at study outcomes, it is striking that 80% of the included studies investigated isolated categories. A more complete picture of autistic individuals’ category learning skills would benefit from a more thorough examination of interrelated categories. Similarly, since the vast majority of studies (64%) were conducted in English, a greater number of studies carried out in other languages would be desirable. These would then permit conclusions about links between category learning and language in autistic individuals.

In sum, results of the current meta-analysis are generally in line with previous syntheses, but specify these. More precisely, autistic persons on average were found not to reach the level of category learning typically achieved by nonautistic individuals; yet the size of the total effect alongside examinations of publication bias indicated that the group difference might be smaller than suggested by earlier overviews. Beyond this, the present results prompt several areas for future research: Firstly, investigation of moderator variables elucidating heterogeneity, for instance, type of mental representation in combination with task characteristics; secondly, looking at downstream effects of suboptimal category learning skills, for example, for academic performance; and thirdly, developing and implementing interventions tailored to the needs of autistic individuals.

## Data Availability

All meta-analytic data and research materials (including our coding scheme) are available online (https://osf.io/gtj2p/).

## Footnotes

1 We note that Demetriou et al. (2018) in their meta-analysis of executive functioning in autism included concept formation. As this was defined as “capacity to shift between mental processes to form new concepts and identify the conceptual relationships shared by stimuli” (Demetriou et al., 2018, supplementary materials), what was termed concept formation here seems to be more akin to cognitive flexibility/set shifting than to category learning in the narrower sense. Hence, we do not regard Demetriou et al.’s work to deal with category learning as such.

## Code availability

The analysis code is available online (https://osf.io/gtj2p/).

This meta-analysis was not preregistered.

## References

[CR1] Appelbaum M, Cooper H, Kline RB, Mayo-Wilson E, Nezu AM, Rao SM (2018). Journal article reporting standards for quantitative research in psychology: The APA publications and communications board task force report. American Psychologist.

[CR2] Ashby FG, Alfonso-Reese LA, Turken AU, Waldron EM (1998). A neuropsychological theory of multiple systems in category learning. Psychological Review.

[CR3] Ashby FG, Gott RE (1988). Decision rules in the perception and categorization of multidimensional stimuli. Journal of Experimental Psychology: Learning, Memory, and Cognition.

[CR4] Ashby FG, Maddox WT (2005). Human category learning. Annual Review of Psychology.

[CR5] Belsley DA, Kuh E, Welsch RE (1980). *Regression diagnostics: Identifying influential data and sources of collinearity*.

[CR6] Billeiter KB, Froiland JM (2023). Diversity of intelligence is the norm within the autism spectrum: Full scale intelligence scores among children with ASD. Child Psychiatry & Human Development.

[CR7] Bishop, D. V. M., Thompson, J., & Parker, A. J. (2022). Can we shift belief in the ‘law of small numbers’? *Royal Society Open Science*, *9*(3). *Article, 211028*. 10.1098/rsos.211028 PMC8889191 35316946

[CR8] Borenstein, M. (Ed.). (2009). In *Introduction to meta-analysis*. John Wiley & Sons.

[CR9] Bott L, Brock J, Brockdorff N, Boucher J, Lamberts K (2006). Perceptual similarity in autism. Quarterly Journal of Experimental Psychology.

[CR10] Bowerman M, Levinson SC (2001). *Language acquisition and conceptual development*.

[CR11] Brooks LR, Rosch E, Lloyd BB (1978). Nonanalytic concept formation and memory for instances. *Cognition and categorization*.

[CR12] Brown J, Aczel B, Jiménez L, Kaufman SB, Grant KP (2010). Intact implicit learning in autism spectrum conditions. Quarterly Journal of Experimental Psychology.

[CR13] Bruner JS, Goodnow J, Austin G (1956). *A study of thinking*.

[CR14] Carmo JC, Souza C, Gonçalves F, Pinho S, Filipe CN, Lachmann T (2017). Effects of categorical representation on visuospatial working memory in autism spectrum disorder. Journal of Clinical and Experimental Neuropsychology.

[CR15] Carter EC, Schönbrodt FD, Gervais WM, Hilgard J (2019). Correcting for bias in psychology: A comparison of meta-analytic methods. Advances in Methods and Practices in Psychological Science.

[CR16] Church BA, Krauss MS, Lopata C, Toomey JA, Thomeer ML, Coutinho MV, Volker MA, Mercado E (2010). Atypical categorization in children with high-functioning autism spectrum disorder. Psychonomic Bulletin & Review.

[CR17] Constable PA, Ring M, Gaigg SB, Bowler DM (2018). Problem-solving styles in autism spectrum disorder and the development of higher cognitive functions. Autism.

[CR18] Cook RD (1977). Detection of influential observation in linear regression. Technometrics.

[CR19] *Corbett, J. E., Venuti, P., & Melcher, D. (2016). Perceptual averaging in individuals with autism spectrum disorder. *Frontiers in Psychology, 7*. 10.3389/fpsyg.2016.01735 PMC5097930 27872602

[CR20] Daniels J, Haber N, Voss C, Schwartz J, Tamura S, Fazel A, Kline A, Washington P, Phillips J, Winograd T, Feinstein C, Wall D (2018). Feasibility testing of a wearable behavioral aid for social learning in children with autism. Applied Clinical Informatics.

[CR21] Demetriou EA, Lampit A, Quintana DS, Naismith SL, Song YJC, Pye JE, Hickie I, Guastella AJ (2018). Autism spectrum disorders: A meta-analysis of executive function. Molecular Psychiatry.

[CR22] Desaunay P, Briant AR, Bowler DM, Ring M, Gérardin P, Baleyte J-M, Guénolé F, Eustache F, Parienti J-J, Guillery-Girard B (2020). Memory in autism spectrum disorder: A meta-analysis of experimental studies. Psychological Bulletin.

[CR23] Dovgopoly A, Mercado E (2013). A connectionist model of category learning by individuals with high-functioning autism spectrum disorder. Cognitive, Affective, & Behavioral Neuroscience.

[CR24] Egger, M., Smith, G. D., Schneider, M., & Minder, C. (1997). Bias in meta-analysis detected by a simple, graphical test. *BMJ, 315*(7109), 629–634. 10.1136/bmj.315.7109.629 PMC2127453 9310563

[CR25] Field C, Allen ML, Lewis C (2016). Are children with autism spectrum disorder initially attuned to object function rather than shape for word learning?. Journal of Autism and Developmental Disorders.

[CR26] *Froehlich, A. L. (2008). *Categorization in autism*. University of Utah.

[CR27] Froehlich AL, Anderson JS, Bigler ED, Miller JS, Lange NT, DuBray MB, Cooperrider JR, Cariello A, Nielsen JA, Lainhart JE (2012). Intact prototype formation but impaired generalization in autism. Research in Autism Spectrum Disorders.

[CR28] Gastgeb HZ, Dundas EM, Minshew NJ, Strauss MS (2012). Category formation in autism: Can individuals with autism form categories and prototypes of dot patterns?. Journal of Autism and Developmental Disorders.

[CR29] Gastgeb HZ, Rump KM, Best CA, Minshew NJ, Strauss MS (2009). Prototype formation in autism: Can individuals with autism abstract facial prototypes?. Autism Research.

[CR30] Gastgeb HZ, Wilkinson DA, Minshew NJ, Strauss MS (2011). Can individuals with autism abstract prototypes of natural faces?. Journal of Autism and Developmental Disorders.

[CR31] Goldstone RL (1996). Isolated and interrelated concepts. Memory & Cognition.

[CR32] Hahn ER, Cantrell L (2012). The shape-bias in Spanish-speaking children and its relationship to vocabulary. Journal of Child Language.

[CR33] Harrer M (2022). *Doing meta-analysis with R: A hands-on guide*.

[CR34] Hartley C, Allen ML (2014). Brief report: Generalisation of word–picture relations in children with autism and typically developing children. Journal of Autism and Developmental Disorders.

[CR35] Hetzroni OE, Hessler M, Shalahevich K (2019). Learning new relational categories by children with autism spectrum disorders, children with typical development and children with intellectual disabilities: Effects of comparison and familiarity on systematicity. Journal of Intellectual Disability Research.

[CR36] Hetzroni OE, Shalahevich K (2018). Structure mapping in autism spectrum disorder: Levels of information processing and relations to executive functions. Journal of Autism and Developmental Disorders.

[CR37] Hoffmann WL, Prior MR (1982). Neuropsychological dimensions of autism in children: A test of the hemispheric dysfunction hypothesis. Journal of Clinical Neuropsychology.

[CR38] Hox, J., Moerbeek, M., & van de Schoot, R. (2002). Multilevel analysis. *Routledge.*

[CR39] Jarrold C, Brock J (2004). To match or not to match? Methodological issues in autism-related research. Journal of Autism and Developmental Disorders.

[CR40] Jimenez, B., Root, J., Shurr, J., & Bouck, E. C. (2021). Using the four stages of learning to assess, set goals, and instruct. *Teaching Exceptional Children, 0*(0) Advance online publication.

[CR41] Kado Y, Sanada S, Oono S, Ogino T, Nouno S (2020). Children with autism spectrum disorder comorbid with attention-deficit/hyperactivity disorder examined by the Wisconsin card sorting test: Analysis by age-related differences. Brain and Development.

[CR42] Kado Y, Sanada S, Yanagihara M, Ogino T, Ohno S, Watanabe K, Nakano K, Morooka T, Oka M, Ohtsuka Y (2012). Executive function in children with pervasive developmental disorder and attention-deficit/hyperactivity disorder assessed by the Keio version of the Wisconsin card sorting test. Brain and Development.

[CR43] Kaland N, Smith L, Mortensen EL (2008). Brief report: Cognitive flexibility and focused attention in children and adolescents with Asperger syndrome or high-functioning autism as measured on the computerized version of the Wisconsin card sorting test. Journal of Autism and Developmental Disorders.

[CR44] Katusic, M. Z., Myers, S. M., Weaver, A. L., & Voigt, R. G. (2021). IQ in autism spectrum disorder: A population-based birth cohort study. *Pediatrics*, *148*(6), article e2020049899. 10.1542/peds.2020-049899 34851412

[CR45] Klinger LG, Dawson G (2001). Prototype formation in autism. Development and Psychopathology.

[CR46] Kruschke, J. K. (2005). Category learning. In K. Lamberts & R. Goldstone (Eds.), *Handbook of cognition* (pp. 184–202). SAGE Publications.

[CR47] Light RJ, Pillemer DB (1984). *Summing up: The science of reviewing research*.

[CR48] Lipsey, M. W., & Wilson, D. B. (2001). Practical meta-analysis. *SAGE* Publications.

[CR49] *Mançe Çalişir, Ö. M., Atbaşoğlu, E. C., Devrimci Özgüven, H., & Ölmez, Ş. (2018). Cognitive features of high-functioning adults with autism and schizophrenia spectrum disorders. *Turkish Journal of Psychiatry*, *29*(1), 1–10. 29730869

[CR50] Markman AB, Ross BH (2003). Category use and category learning. Psychological Bulletin.

[CR51] Maule J, Stanworth K, Pellicano E, Franklin A (2017). Ensemble perception of color in autistic adults. Autism Research.

[CR52] McGregor KK, Bean A (2012). How children with autism extend new words. Journal of Speech, Language, and Hearing Research.

[CR53] Medin DL, Lynch EB, Solomon KO (2000). Are there kinds of concepts?. Annual Review of Psychology.

[CR54] Medin DL, Rips LJ, Holyoak KJ, Morrison RG (2005). Concepts and categories: Memory, meaning, and metaphysics. *The Cambridge handbook of thinking and reasoning*.

[CR55] Mercado E, Chow K, Church BA, Lopata C (2020). Perceptual category learning in autism spectrum disorder: Truth and consequences. Neuroscience & Biobehavioral Reviews.

[CR56] Meyer AT (2014). *Visually guided prototype learning in children with autism spectrum disorders*.

[CR57] Milton D, Jordan R, Roberts JM, Hume K (2019). Difference versus disability: Implications of characterisation of autism for education and support. *The SAGE handbook of autism and education*.

[CR58] Minshew NJ, Goldstein G, Siegel DJ (1997). Neuropsychologic functioning in autism: Profile of a complex information processing disorder. Journal of the International Neuropsychological Society.

[CR59] Minshew NJ, Meyer J, Goldstein G (2002). Abstract reasoning in autism: A disassociation between concept formation and concept identification. Neuropsychology.

[CR60] Molesworth CJ, Bowler DM, Hampton JA (2005). The prototype effect in recognition memory: Intact in autism?. Journal of Child Psychology and Psychiatry.

[CR61] Molesworth CJ, Bowler DM, Hampton JA (2008). When prototypes are not best: Judgments made by children with autism. Journal of Autism and Developmental Disorders.

[CR62] Molesworth C, Chevallier C, Happé F, Hampton JA (2015). Children with autism do not show sequence effects with auditory stimuli. Journal of Experimental Psychology: General.

[CR63] Mottron L (2004). Matching strategies in cognitive research with individuals with high-functioning autism: Current practices, instrument biases, and recommendations. Journal of Autism and Developmental Disorders.

[CR64] Mottron L, Dawson M, Soulières I, Hubert B, Burack J (2006). Enhanced perceptual functioning in autism: An update, and eight principles of autistic perception. Journal of Autism and Developmental Disorders.

[CR65] *Nader, A.-M., Tullo, D., Bouchard, V., Degré-Pelletier, J., Bertone, A., Dawson, M., & Soulières, I. (2022). Category learning in autism: Are some situations better than others? *Journal of Experimental Psychology: General*, *151*(3), 578–596. 10.1037/xge0001092 34582232

[CR66] Naigles LR, Kelley E, Troyb E, Fein D (2013). Residual difficulties with categorical induction in children with a history of autism. Journal of Autism and Developmental Disorders.

[CR67] O’Riordan M, Plaisted K (2001). Enhanced discrimination in autism. The Quarterly Journal of Experimental Psychology Section A.

[CR68] Park S, Park J-E, Cho S-C, Kim B-N, Shin M-S, Kim J-W, Cho IH, Kim SA, Park M, Park T-W, Son J-W, Chung U-S, Yoo HJ (2014). No association of the norepinephrine transporter gene (SLC6A2) and cognitive and behavioural phenotypes of patients with autism spectrum disorder. European Archives of Psychiatry and Clinical Neuroscience.

[CR69] Patry MB, Horn EM (2019). Schema development in individuals with autism: A review of the literature. Review Journal of Autism and Developmental Disorders.

[CR70] Pellicano E, Burr D (2012). When the world becomes ‘too real’: A Bayesian explanation of autistic perception. Trends in Cognitive Sciences.

[CR71] Plaisted K, O’Riordan M, Baron-Cohen S (1998). Enhanced discrimination of novel, highly similar stimuli by adults with autism during a perceptual learning task. Journal of Child Psychology and Psychiatry.

[CR72] Posner MI, Keele SW (1968). On the genesis of abstract ideas. Journal of Experimental Psychology.

[CR73] Posner MI, Keele SW (1970). Retention of abstract ideas. Journal of Experimental Psychology.

[CR74] *Potrzeba, E. R., Fein, D., & Naigles, L. (2015). Investigating the shape bias in typically developing children and children with autism spectrum disorders. *Frontiers in Psychology*, *06*. 10.3389/fpsyg.2015.00446 PMC4404809 25954219

[CR75] *Powell, P. (2016). Cognitive aging in autism spectrum disorder. *The University of North Carolina at Chapel Hill University Libraries.* 10.17615/DQV5-N916.

[CR76] Powell PS, Klinger LG, Klinger MR (2017). Patterns of age-related cognitive differences in adults with autism spectrum disorder. Journal of Autism and Developmental Disorders.

[CR77] Rosch E, Mervis CB (1975). Family resemblances: Studies in the internal structure of categories. Cognitive Psychology.

[CR78] Rumsey JM (1985). Conceptual problem-solving in highly verbal, nonretarded autistic men. Journal of Autism and Developmental Disorders.

[CR79] Sandbank M, Bottema-Beutel K, Crowley S, Cassidy M, Dunham K, Feldman JI, Crank J, Albarran SA, Raj S, Mahbub P, Woynaroski TG (2020). Project AIM: Autism intervention meta-analysis for studies of young children. Psychological Bulletin.

[CR80] Sapey-Triomphe L-A, Sonié S, Hénaff M-A, Mattout J, Schmitz C (2018). Adults with autism tend to undermine the hidden environmental structure: Evidence from a visual associative learning task. Journal of Autism and Developmental Disorders.

[CR81] Schipul SE, Just MA (2016). Diminished neural adaptation during implicit learning in autism. NeuroImage.

[CR82] Schipul SE, Williams DL, Keller TA, Minshew NJ, Just MA (2012). Distinctive neural processes during learning in autism. Cerebral Cortex.

[CR83] Shu B-C, Lung F-W, Tien AY, Chen B-C (2001). Executive function deficits in non-retarded autistic children. Autism.

[CR84] Sloutsky, V. M., & Deng, W. (Sophia). (2019). Categories, concepts, and conceptual development. *Language, Cognition and Neuroscience, 34*(10), 1284–1297. 10.1080/23273798.2017.1391398 PMC7410261 32775486

[CR85] Solomon M, Buaminger N, Rogers SJ (2011). Abstract reasoning and friendship in high functioning preadolescents with autism spectrum disorders. Journal of Autism and Developmental Disorders.

[CR86] Soulières I, Mottron L, Giguère G, Larochelle S (2011). Category induction in autism: Slower, perhaps different, but certainly possible. Quarterly Journal of Experimental Psychology.

[CR87] Tager-Flusberg H (2004). Strategies for conducting research on language in autism. Journal of Autism and Developmental Disorders.

[CR88] Tecoulesco L, Fein D, Naigles LR (2021). What categorical induction variability reveals about typical and atypical development. Journal of Child Language.

[CR89] *Tovar, Á. E., Rodríguez-Granados, A., & Arias-Trejo, N. (2020). Atypical shape bias and categorization in autism: Evidence from children and computational simulations. *Developmental Science*, *23*(2). 10.1111/desc.12885 31271684

[CR90] Tversky A, Kahneman D (1971). Belief in the law of small numbers. Psychological Bulletin.

[CR91] van Boxtel, J. J. A., & Lu, H. (2013). A predictive coding perspective on autism spectrum disorders. *Frontiers in Psychology, 4*. 10.3389/fpsyg.2013.00019 PMC3556598 23372559

[CR92] Van de Cruys S, Evers K, Van der Hallen R, Van Eylen L, Boets B, de-Wit L, Wagemans J (2014). Precise minds in uncertain worlds: Predictive coding in autism. Psychological Review.

[CR93] Vanpaemel W, Bayer J (2021). Prototype-based category learning in autism: A review. Neuroscience and Biobehavioral Reviews.

[CR94] Viechtbauer W (2010). Conducting meta-analyses in *R* with the metafor package. Journal of Statistical Software.

[CR95] Vladusich T, Olu-Lafe O, Kim D-S, Tager-Flusberg H, Grossberg S (2010). Prototypical category learning in high-functioning autism. Autism Research.

[CR96] Williams DL, Mazefsky CA, Walker JD, Minshew NJ, Goldstein G (2014). Associations between conceptual reasoning, problem solving, and adaptive ability in high-functioning autism. Journal of Autism and Developmental Disorders.

[CR97] Williams DL, Minshew NJ, Goldstein G (2015). Further understanding of complex information processing in verbal adolescents and adults with autism spectrum disorders. Autism.

[CR98] Wolff N, Stroth S, Kamp-Becker I, Roepke S, Roessner V (2022). Autism spectrum disorder and IQ—A complex interplay. Frontiers in Psychiatry.

[CR99] World Health Organization (2019). *International statistical classification of diseases and related health problems* (11th ed.). 10.2471/BLT.25.294650 PMC13531092 42683092

[CR100] Wright K, Kelley E, Poulin-Dubois D (2016). Biological motion and the animate–inanimate distinction in children with high-functioning autism spectrum disorder. Research in Autism Spectrum Disorders.

[CR101] Zeidan J, Fombonne E, Scorah J, Ibrahim A, Durkin MS, Saxena S, Yusuf A, Shih A, Elsabbagh M (2022). Global prevalence of autism: A systematic review update. Autism Research.

[CR102] Zettersten M, Lupyan G (2020). Finding categories through words: More nameable features improve category learning. Cognition.

